# Editorial for “Advances in Oral Diseases Diagnosis and Management: 2nd Edition”

**DOI:** 10.3390/diagnostics15121454

**Published:** 2025-06-07

**Authors:** Dimitris Tatsis, Konstantinos Paraskevopoulos

**Affiliations:** Department of Oral & Maxillofacial Surgery, Aristotle University of Thessaloniki, Specialized Cancer Treatment and Reconstruction Centre, General Hospital of Thessaloniki “George Papanikolaou”, 57010 Thessaloniki, Greece; kostparas@yahoo.gr

The field of oral disease diagnosis and management has undergone significant transformation in recent years, propelled by innovations in diagnostic technologies, molecular biology, and interdisciplinary clinical approaches. Oral diseases, ranging from periodontal disease to oral cancers, pose a major global health challenge due to their prevalence and impact on patients’ quality of life [1]. The second edition of the Special Issue, titled “Advances in Oral Diseases Diagnosis and Management”, published in *Diagnostics*, builds on the success of its predecessor by presenting innovative research that addresses critical gaps in the early detection, diagnosis, and treatment of these conditions.

Recent advancements in oral diagnostics have leveraged tools such as high-resolution imaging, biomarker profiling, and artificial intelligence-driven analytics to enable earlier and more precise detection of oral pathologies [2]. However, challenges remain, including the need for standardized diagnostic protocols across diverse populations, a deeper understanding of molecular mechanisms underlying disease progression, and the development of targeted therapies for complex conditions such as oral squamous cell carcinoma [1,3]. This Special Issue addresses these gaps by highlighting studies that integrate novel diagnostic methodologies, therapeutic strategies, and research into host–microbiome interactions to advance patient-centered care.

The contributions to this Special Issue highlight a range of innovative approaches. For instance, a retrospective study on pathological fractures of the mandible explored the challenges of managing compromised bone quality due to conditions like osteoradionecrosis and medication-related osteonecrosis, offering applied insights into multidisciplinary treatment strategies [4]. Another investigation utilized mucoscopy as a non-invasive tool to assess oral lichen planus, demonstrating its potential to differentiate inflammatory conditions and enhance diagnostic accuracy [5]. Additionally, the application of targeted optical coherence tomography (OCT) in oral cancer diagnosis has shown promise for evaluating tissue microstructure, paving the way for standardized protocols [6]. These works, alongside broader research on microbial dynamics and clinical interventions, underscore the importance of integrating advanced diagnostics with clinical practice [7,8].

Beyond the Special Issue, the recent literature has emphasized the interplay between oral and systemic health. Studies have explored the efficacy of interventions such as sodium hypochlorite mouthwash for periodontal disease management [8], as well as the association between oral health and broader disease outcomes [2]. These findings highlight the need for holistic diagnostic and management strategies that bridge oral and systemic health.

Looking to the future, several research priorities emerge. Standardizing diagnostic criteria across global populations is essential to ensure equitable access to early detection and treatment [1]. The integration of AI into clinical workflows requires robust, multicenter validation studies to confirm its reliability and applicability. Furthermore, personalized therapies informed by genetic and epigenetic profiling could transform the management of oral cancers and autoimmune disorders. Interdisciplinary collaboration across oral pathology, maxillofacial surgery, and molecular biology will be critical to translating these innovations into clinical practice.

This Special Issue serves as both a milestone in current research and a call to action for future exploration. We hope it will inspire researchers, clinicians, and policymakers to address the remaining challenges in oral disease diagnosis and management, ultimately improving patient outcomes on a broader scale.

We extend our gratitude to the authors, reviewers, and editorial team for their dedication to this Special Issue. We invite readers to engage with the research presented here and to contribute to the ongoing advancement of this vital field.
